# Monitoring the patient under mechanical ventilation: a Brazilian survey

**DOI:** 10.1186/cc14702

**Published:** 2015-09-28

**Authors:** Alexandre M Isola, Eliana B Caser, Ricardo G Rodrigues, Carmen SV Barbas

**Affiliations:** 1 Grupo de Assistência Ventilatória, Universidade de São Paulo, AvBrooklyn Novo, São Paulo, SP, Brazil

## Introduction

Adequate monitoring of the patient on mechanical ventilation (IMV) reduces complications and improves safety. In order to know more about this important issue in Brazil, we performed an electronic survey.

## Methods

An open web-based electronic survey with questions about mechanical ventilation became available for intensive care practitioners in Brazil (DATAVENT) from 17 November 2013 to 31 August 2014 regarding the following questions: 1. Which of these predictive indexes or maneuvers do you (or someone in your ICU staff) routinely apply during the IMV withdrawal process? 2. Check what of these parameters are measured or calculated in your ICU daily practice? 3. How do you obtain the driving pressure value? 4. How do you obtain the resistive pressure value? 5. Check what are the imaging resources available in your hospital for the patient under IMV? 6. What is the value of tidal volume (ml/kg of predicted body weight (PBW)) that you use in patients under IMV without lung disease, including immediate postoperative period?

## Results

There were 418 responders. Two hundred and ninety-seven of the intensive care practitioners (71.05 %) use SpO_2 _as an index in withdrawal process. Two hundred and seventy-four (65.55 %) use the rapid shallow breathing index. One hundred and five (25.12 %) use the maximal inspiratory pressure (PiMax) and 32 (7.66 %) use the integrative weaning index (IWI). In total, 336 (80.38 %) measure plateau pressure, 295 (70.57 %) measure intrinsic PEEP, 243 (58.13 %) measure static compliance, 99 (23.68 %) measure driving pressure and 98 (23.44 %) measure resistive pressure as routine. Thirty-eight (9.09 %) have access to monitor work of breath (WOB). A total of 216 (51.67 %) does not know how to obtain the driving pressure. A total of 241 (57.65 %) answered correctly how to obtain resistive pressure. A total of 329 (78.71 %) has access to conventional computerized tomography in their hospitals. A total of 245 (58.61 %) has ultrasound and 223 (53.35 %) have echocardiography available. A total of 246 (58.85 %) perform chest radiography on a daily basis. A total of 183 (43.77 %) keeps the tidal volume of a patient under IMV without lung diseases (including postoperative patients) in 8 ml/kg/PBW. In total, 139 (33.25 %) use 6 ml/kg/PBW, 68 (16.26 %) use 7 ml/kg/PBW, 14 (3.35 %) use 9-10 ml/kg/PBW and 14 (3.35 %) use less than 6 ml/kg/PBW.

## Conclusion

Monitoring of the patient under IMV is realized, focusing on the withdrawing process and basic mechanical parameters. Practitioners need to know more about obtaining the driving pressure and its important safety value, aiming to improve its use in their daily practice. Image resources availability on the ICU can be improved, mainly echography resources. The use of a tidal volume of 6 ml/kg/PBW in patients without lung disease should be encouraged.

